# Identifying and correcting epigenetics measurements for systematic sources of variation

**DOI:** 10.1186/s13148-018-0471-6

**Published:** 2018-03-21

**Authors:** Flavie Perrier, Alexei Novoloaca, Srikant Ambatipudi, Laura Baglietto, Akram Ghantous, Vittorio Perduca, Myrto Barrdahl, Sophia Harlid, Ken K. Ong, Alexia Cardona, Silvia Polidoro, Therese Haugdahl Nøst, Kim Overvad, Hanane Omichessan, Martijn Dollé, Christina Bamia, José Marìa Huerta, Paolo Vineis, Zdenko Herceg, Isabelle Romieu, Pietro Ferrari

**Affiliations:** 1 Nutritional Methodology and Biostatistics Group, International Agency for Research on Cancer (IARC), World Health Organization, 150 cours Albert Thomas, 69372 Lyon CEDEX 08, France; 2 Epigenetics Group, IARC, Lyon, France; 3 Department of Clinical and Experimental Medicine, University of Pisa, Pisa, Italy; 4 MAP5 – UMR CNRS 8145, Université Paris Descartes, Sorbonne Paris Cité, Paris, France; 5 Division of Cancer Epidemiology, German Cancer Research Center (DKFZ), Heidelberg, Germany; 6 Department of Radiation Sciences, Oncology, Umeå University, Umeå, Sweden; 7 MRC Epidemiology Unit, Institute of Metabolic Science, University of Cambridge School of Clinical Medicine, Cambridge, UK; 8 IIGM – Italian Institute for Genomic Medicine, Torino, Italy; 9 Department of Community Medicine, UiT - The Arctic University of Norway, Tromsø, Norway; 10 Section for Epidemiology, Department of Public Health, Aarhus University, Aarhus, Denmark; 11 Department of Cardiology, Aalborg University Hospital, Aalborg, Denmark; 12 CESP, Fac. de médecine - Univ. Paris-Sud, Fac. de médecine – UVSQ, INSERM, Université Paris-Saclay, Villejuif, France; 13 Gustave Roussy, Villejuif, France; 14 Centre for Health Protection (pb12), National Institute of Public Health and the Environment (RIVM), Bilthoven, Netherlands; 15 Hellenic Health Foundation, Athens, Greece; 16 WHO Collaborating Center for Nutrition and Health, Unit of Nutritional Epidemiology and Nutrition in Public Health, Department of Hygiene, Epidemiology and Medical Statistics, School of Medicine, National and Kapodistrian University of Athens, Athens, Greece; 17 Department of Epidemiology, Murcia Regional Health Council, IMIB-Arrixaca, Murcia, Spain; 18 CIBER Epidemiología y Salud Pública (CIBERESP), Madrid, Spain; 19 MRC/PHE Centre for Environment and Health, School of Public Health, Imperial College London, London, UK; 20 Nutritional Epidemiology Group, IARC, Lyon, France

**Keywords:** Epigenetics, PC-PR2, Normalization, Methylation, Smoking status

## Abstract

**Background:**

Methylation measures quantified by microarray techniques can be affected by systematic variation due to the technical processing of samples, which may compromise the accuracy of the measurement process and contribute to bias the estimate of the association under investigation. The quantification of the contribution of the systematic source of variation is challenging in datasets characterized by hundreds of thousands of features.

In this study, we introduce a method previously developed for the analysis of metabolomics data to evaluate the performance of existing normalizing techniques to correct for unwanted variation. Illumina Infinium HumanMethylation450K was used to acquire methylation levels in over 421,000 CpG sites for 902 study participants of a case-control study on breast cancer nested within the EPIC cohort. The principal component partial R-square (PC-PR2) analysis was used to identify and quantify the variability attributable to potential systematic sources of variation. Three correcting techniques, namely ComBat, surrogate variables analysis (SVA) and a linear regression model to compute residuals were applied. The impact of each correcting method on the association between smoking status and DNA methylation levels was evaluated, and results were compared with findings from a large meta-analysis.

**Results:**

A sizeable proportion of systematic variability due to variables expressing ‘batch’ and ‘sample position’ within ‘chip’ was identified, with values of the partial R^2^ statistics equal to 9.5 and 11.4% of total variation, respectively. After application of ComBat or the residuals’ methods, the contribution was 1.3 and 0.2%, respectively. The SVA technique resulted in a reduced variability due to ‘batch’ (1.3%) and ‘sample position’ (0.6%), and in a diminished variability attributable to ‘chip’ within a batch (0.9%). After ComBat or the residuals’ corrections, a larger number of significant sites (*k* = 600 and *k* = 427, respectively) were associated to smoking status than the SVA correction (*k* = 96).

**Conclusions:**

The three correction methods removed systematic variation in DNA methylation data, as assessed by the PC-PR2, which lent itself as a useful tool to explore variability in large dimension data. SVA produced more conservative findings than ComBat in the association between smoking and DNA methylation.

**Electronic supplementary material:**

The online version of this article (10.1186/s13148-018-0471-6) contains supplementary material, which is available to authorized users.

## Background

Epigenetics aims at investigating changes in gene activity not attributable to changes in the DNA sequence [1]. An increasing number of studies analysed epigenetics in relation to modifiable environmental exposures of epidemiologic interest, such as smoking [2–4], alcohol consumption [5], maternal plasma folate [6] and other vitamin involved in the one carbon metabolism pathway [7], as well as the role of epigenetic profiles on the risk of developing chronic diseases, including cancer [8]. DNA methylation is a mechanism of epigenetic regulation that involves the addition of methyl groups (–CH3) to the cytosine of a cytosine-guanine DNA sequence. DNA methylation level at one CpG site is frequently expressed as the percentage of cells that are methylated at that specific site. The Illumina Infinium HumanMethylation450K BeadChip (HM450K) quantifies DNA methylation at more than 450,000 interrogated CpG sites, expressing methylation level as the ratio of the methylated probe intensity to the overall intensity, which is the sum of the methylated and unmethylated probe intensities [9].

Methylation levels are influenced by many factors including aging [10] and environmental exposure [11, 12], but might also be affected by systematic variation due to the processing of the biospecimens, e.g. variability attributed to batch (a sub-group of samples processed at the same time, 96 samples per batch in the HM450K), chip position within batches (8 chips per batch in the HM450K) and the position of the samples within the chip [13]. Methods of correcting for the sources of methylation variability include ComBat, based on an empirical Bayes method [14] and the surrogate variables analysis (SVA) [15, 16]. An alternative method consists in the computation of residuals from a beta regression, where methylation levels were regressed on the major sources of methylation variability.

The large dimension of new generation methylation arrays makes it difficult to quantify the amount of variability attributable to systematic sources of variation. The principal component partial R-square (PC-PR2) method was developed to quantify the contribution of sources of variation defined a priori in large dimensional data [17].

Smoking exposure has been analysed in many studies [2–4], which offers a large comparative pool of results. Smoking has also been shown to have a major impact on the epigenome and hence provides a large number of significant CpGs to analyse. For these reasons, in this work, we have chosen to evaluate the performance of ComBat, SVA and the residuals’ method to correct for potential systematic variability in methylation measurements, in the association between smoking and DNA methylation levels from DNA samples of subjects of a nested case-control study on breast cancer conducted within the European Prospective Investigation into Cancer and nutrition (EPIC) study. The PC-PR2 method was used to quantify the extent of total epigenetics variability before and after applying each correcting method.

## Methods

### Study population

The EPIC study [18, 19] is a multicentre study that recruited over 521,000 study participants, between 1992 and 2000 in 23 regional or national centres in 10 European countries (Denmark, France, Germany, Greece, Italy, Netherlands, Norway, Spain, Sweden and the UK). Among the 367,903 women recruited in EPIC, we excluded 19,583 participants with prevalent cancers at recruitment (except non-melanoma skin cancer) and 2892 women that were lost during follow-up. Malignant primary breast cancer (BC) occurred for 10,713 of them from 1992 to 2010. A nested case-control study was designed among women who completed dietary and lifestyle questionnaires and provided blood samples at recruitment (baseline), which included 3858 invasive BC cases. Each case was matched to a randomly selected control among cancer-free women by recruitment centre and the following baseline variables: age, menopausal status, fasting status, current use of oral contraceptive pill or hormone replacement therapy and time of blood collection [20].

### Genome-wide DNA profiling assessment

Genome-wide DNA-methylation profiles in buffy coat samples was quantified using the Illumina Infinium HumanMethylation450K (HM450K) BeadChip assay [9] in 960 biospecimens of women included in the BC nested case-control study [21]. The 480 cases were selected based on estrogen receptor status and by selecting equal proportions of subjects with above or below median level of dietary folate. Matched controls were the same than those selected for the whole study. A total of 20 biospecimens with replicates were used to compare technical inter- and intra-assay batch effects and then excluded from the main analysis. We also excluded 19 matched pairs where at least one of the two samples had a low-quality bisulfite conversion efficiency (intensity signal < 4000) or which did not pass all the Illumina GenomeStudio quality control steps, which were based on built-in control probes for staining, hybridization, extension, and specificity [22]. A total of 451 completed matched pairs (*n* = 902) were retained for the main statistical analyses. In any given sample, probes with detection *p* value higher than 0.05 were assigned ‘missing’ status. After the exclusion of 14,548 cross-reactive probes, 47,963 probes overlapping known SNPs with minor allele frequency (MAF) of ≥ 5% in the overall population (European ancestry) [23] and 1483 low-quality probes (missing in more than 5% of the samples), 421,583 probes were included in the statistical analyses.

For each probe, *β* value was calculated as the ratio of methylated intensity and the overall intensity, defined as the sum of methylated and unmethylated intensities. The following preliminary adjustment steps were applied to the *β* values: (i) color bias normalization using smooth quantile normalization to correct for the two color channels; (ii) quantile normalization [24]; (iii) type I and type II bias correction using the beta-mixture quantile normalization (BMIQ) [25]. Then, *M* values, defined as ${M}_{\mathrm{values}}={\log}_2\left(\frac{\beta_{\mathrm{values}}}{1-{\beta}_{\mathrm{values}}}\right)$, were computed [26]. In this work, the *β* and *M* values obtained after the preliminary normalization steps were referred to as the raw *β* and *M* values.

The amount of white blood cell counts (T cells (CD8^+^T and CD4^+^T), natural killer (NK) cells, B cells, monocytes and granulocytes) was quantified using Houseman’s estimation method [27]. The percentage of granulocytes was not included in this analysis as it is collinear with the five other white blood cell counts: the total of the percentages of the six leukocyte subtype counts is 1.

**Fig. 1:**
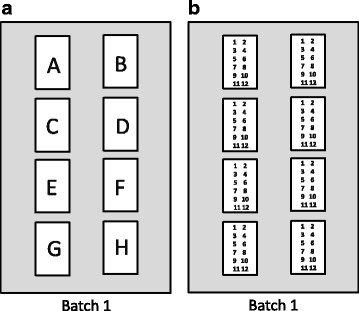
Description of laboratory variables. **a** Position of chips within batches, each batch was made of 8 chips. **b** Sample position within chips, each chip contains 12 samples

For the DNA methylation measurements with the HM450K BeadChip, samples were aliquoted into 10 batches; each batch was made of 8 chips, and each chip contained 12 samples (located in 2 columns of 6 rows). Chip position represented the position of the chips within a batch, as illustrated in Fig. 1a, and sample position represented the position of the samples within a chip, as in Fig. 1b.

### Lifestyle exposures

Data on lifestyle exposures were collected at recruitment through country- or centre-specific dietary and lifestyle questionnaires [18]. Smoking status was categorized into ever (former/current) and never smokers and was not associated to any of the technical covariates.

### Statistical analyses

In order to inspect the variability of DNA methylation levels, we first visually inspected, via box plots, global DNA methylation levels by batch, chip and sample positions. The principal component partial R-square (PC-PR2) method was used to quantify the contribution of laboratory factors and other characteristics of the samples to the between-sample variability observed [17]. First, principal component analysis (PCA) was carried out, by the PC-PR2, on the matrix *X* of epigenetics data of dimension *n* × *p* (*n* = 902: number of study samples and *p* = 421,583: number of probes). In PCA, eigenvalues and eigenvectors are usually obtained from the matrix *X*^′^*X* of dimension *p* × *p*. In this case, and in general with *-omics* data, *p* is very large (*p* ≫ *n*), and the decomposition of *X*^′^*X* can be cumbersome. A particularly appealing procedure consists in extracting eigenvalues and eigenvectors from the matrix *XX*^′^, of dimension *n* × *n* [28], which is way easier to handle, being *n* much smaller than *p*. Once eigenvalues were extracted, the *q* first components explained an amount of total variability in *X* greater than a given threshold, i.e. 80% in this study. Then, each of the *q* first PCA score components was, in turn, linearly regressed on a list of independent covariates (*Z*), comprising of laboratory factors and characteristics of the samples. Values of the partial R^2^ statistics were assessed for each *Z* covariate, separately in each component-specific model [29]. An overall partial R^2^ was computed for each *Z* covariate with a weighted average of their component-specific partial R^2^ using the corresponding *q* eigenvalues as weights, conditional to all other covariates in the model. The covariates that we have entered into the regression include batch, chip position, row sample position, recruitment centre, proportions of leukocyte subtypes (CD8^+^T, CD4^+^T, NK, B cells and monocytes), alcohol consumption (g/day), age (year), BMI (kg/m^2^), menopausal status (post- vs. pre-menopause), smoking (ever vs. never smokers), BC status (case or control) and dietary folate intake (μg/day).

### Removing unwanted variation

To remove the two most important sources of variation identified with the PC-PR2 from DNA methylation levels, three different correcting techniques were applied to raw *β* and *M* values: residuals, ComBat and SVA. The ComBat method [14] is a procedure based on an empirical Bayes approach that can correct only for one covariate at the time. Given the presence of multiple sources of variation, we have applied two parametric ComBat in multiple sequential steps: ComBat was first applied to remove batch variability, and then a second ComBat step was run to remove variability due to row sample position. Methylation *β* values that after the application of ComBat were lower than 0 or larger than 1 were set to 0 and 1 respectively. The surrogate variables analysis (SVA) is a method developed to remove pre-identified sources of variability but also non-known sources of variability, i.e. variability which is not specified in the SVA model, using surrogate variables [15, 16]. Once surrogate variables were assessed by SVA, residuals from a regression modeling methylation level according to the surrogate variables were computed to remove the unwanted variation.

As the *β* values are continuous in the [0,1] interval, the calculation of the residuals for the residuals’ method and SVA method were based on beta regression. To be comparable to the ComBat and raw (i.e. uncorrected) data, residuals computed with the residuals’ and the SVA methods needed to be rescaled as follows:

$$ {res}_{\mathrm{scaled},j}=\frac{{\mathrm{res}}_{\mathrm{raw},j}-\min \left({\mathrm{res}}_{\mathrm{raw},j}\right)}{\max \left({\mathrm{res}}_{\mathrm{raw},j}\right)-\min \left({\mathrm{res}}_{\mathrm{raw},j}\right)}\left(\max\ \left({\mathrm{raw}}_j\right)-\min\ \left({\mathrm{raw}}_j\right)\ \right)+\max\ \left({\mathrm{raw}}_j\right) $$

where *j* = 1…421,583, raw_*j*_ represents the raw *β* values measured in site *j* and res_raw, *j*_ the residuals computed for site *j* before transformation.

In order to check the efficacy of the three correcting techniques, a second PC-PR2 analysis was used to quantify the contribution of each laboratory factor to total variability, after each of the normalization methods.

Same approach was used for *M* values using a linear regression instead of beta regression to compute residuals from the residuals’ and the SVA methods.

In order to compare sample individual values before and after correction, raw and corrected *β* and *M* values of the probe cg00000029 were visually inspected. In this site, in addition to the three tested methods, a second residuals’ method was also computed using random effects instead of fixed affects to remove unwanted variation, from a beta or linear mixed regression, respectively for *β* and *M* values.

### CpG site-specific models

The association between smoking status and each of the 421,583 CpG sites was carried out before and after application of each normalization method. Beta regression models were used for *β* values and linear regression models for *M* values, with adjustment for chip position, recruitment centre, percentages of five leukocyte subtypes, age at recruitment, menopausal status and BC status. The standard adjustment models, i.e. models using the raw methylation values, were also adjusted for batch and row sample position. In order to compare the epigenome-wide distribution of *p* values with the expected null distribution of *p* values, the inflation factor *λ* was computed and the quantile-quantile (QQ) plots were generated. The inflation factor was defined as the ratio of the median of the observed log_10_ transformed *p* values and the median of the expected log_10_ transformed *p* values. False discovery rate (FDR) was used to control for multiple testing. In order to compare the performance of the different correction methods with a nominal reference, the list of *k* significant CpG sites (*q* values < 0.05) associated with smoking was compared to the results of a large meta-analysis carried out in the CHARGE consortium, a recent large meta-analysis on the link between the epigenetic signature of cigarette smoking that pooled data from 16 studies, and included about 16,000 individuals [4]. In CHARGE, smoking status was statistically significantly associated with DNA methylation level (*β* values) in 18,760 sites, after FDR correction of *p* values.

In order to compare the performance of the correction methods, the relative sensitivity and specificity of each correcting method were computed. We considered the CpG sites significantly associated to smoking in the CHARGE consortium as the true positives, i.e. an arbitrary gold standard, given that this is a well-powered reference study and the largest to date.

Preprocessing steps and statistical analysis were carried out using the R software (https://www.r-project.org/) and Bioconductor packages [30], including ‘lumi’ and ‘wateRmelon’ for the adjustment step, ‘sva’ [31] for ComBat and SVA corrections, and ‘betareg’ for beta regression models. The PC-PR2 method was computed using the R code available in Fages et al.’s supplementary material [17].

## Results

**Fig. 2:**
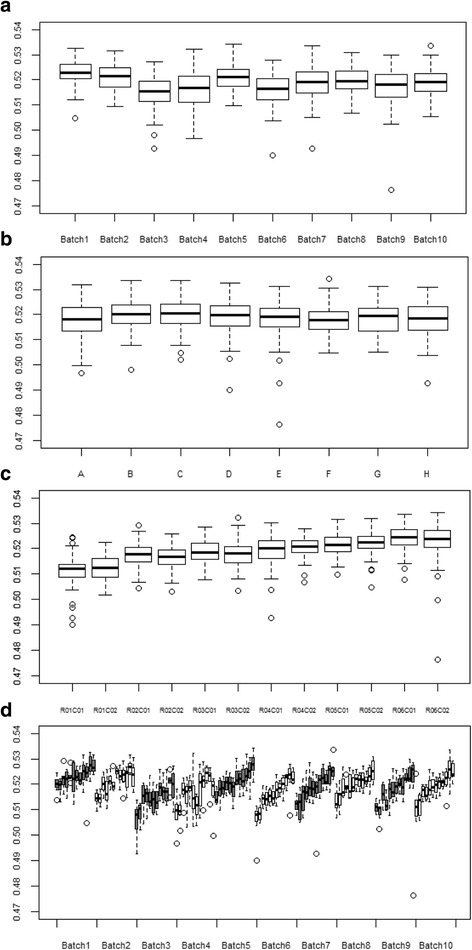
Box plots of global methylation (*β* values) according to laboratory factors. **a** Batch. **b** Chip position within batches. **c** Sample position within chips. **d** Batches and sample position within chips

DNA measurements of the first and the last batches were conducted roughly 3 months apart. DNA measurement of two consecutive batches varied from 3 to 14 days. Box plots of global methylation (i.e. mean of methylation levels in all the CpG sites) showed a random variation of global methylation levels between batches, as reported in Fig. 2a for *β* values. Global methylation between chip positions did not present large variation (Fig. 2b). Sample position within the chip systematically influenced global methylation, with levels by rows, showing a progressive constant increase in methylation, a feature not observed by column, as displayed in Fig. 2c. The impact of row sample position on global methylation was even stronger when batches were evaluated separately (Fig. 2d). Global methylation computed with *M* values gave similar results (Additional file 1: Figure S1).

Tables 1 and 2 show the results of PC-PR2 to quantify the amount of total variability of DNA methylation explained respectively by laboratory factors and characteristics of the samples (recruitment centre, the five percentages of leukocyte subtypes, alcohol intake, age, BMI, menopausal status, smoking, breast cancer status and diet folate intake), for raw *β* and *M* values. Findings were similar for raw *β* and *M* values; the largest contribution to the overall variability came from row sample position and batch explaining, respectively, 11.4 and 9.5% (*β* values), and 12.3 and 9.7% (*M* values) of overall methylation variation. Chip position contributed to 6.5 and 6.8%, for raw *β* and *M* values respectively. The percentages of leukocyte subtypes and centre explained most of the variation of DNA methylation due to sample characteristics for raw *β* and *M* values. Each of the remaining tested other sample characteristics explained less than 0.5% of total variation.

**Table 1:** Values of weighted partial R^2^ (%) from PC-PR2 analysis indicating the proportion of variability of methylation levels, before and after normalization step, explained by a specific set of laboratory factors

Values	Methods^a^	Row sample position	Batch	Chip position	Total^b^
*β* values	Raw	11.4	9.5	6.5	30.4
	Residuals	0.2	1.3	5.9	17.9
	ComBat	0.2	1.3	6.0	17.1
	SVA	0.6	1.3	0.9	6.5
*M* values	Raw	12.3	9.7	6.8	30.7
	Residuals	0.2	1.2	5.8	16.5
	ComBat	0.2	1.3	6.2	17.0
	SVA	0.4	0.7	0.8	5.3

^a^Residuals, COMBAT and SVA methods used to correct effect due to batch and row sample position (within the chips)

^b^Total variability explained by laboratory factors and characteristics of the samples (recruitment centre, the five percentages of leukocyte subtypes, alcohol consumption, age and BMI, menopausal status, smoking, BC status and dietary folate)

**Table 2:** Values of weighted partial R^2^ (%) from PC-PR2 analysis indicating the proportion of variability of raw methylation levels explained by a specific set of covariates

Characteristics of samples	*β* values	*M* values
Recruitment centre	3.0	2.9
Percentages of leukocyte subtypes		
CD4T	3.2	3.2
CD8T	3.7	3.1
Natural killers	5.2	4.7
B cells	1.7	1.1
Monocytes	0.4	0.4
Alcohol intake at recruitment	0.2	0.1
Age at recruitment	0.4	0.4
BMI at recruitment	0.1	0.1
Menopausal status	0.2	0.2
Smoking status	0.1	0.2
Breast cancer status	0.1	0.1
Dietary folate	0.1	0.1

### Removing unwanted variation

All the three correcting methods decreased the contribution of row position and batch to similar neglectable levels, whereas only SVA appeared to reduce the contribution to variability due to chip position (Table 1). The amount of variability explained by laboratory factors and sample characteristics for raw *β* values decreased from 30.4 to 17.9% and 17.1% using, respectively, the residuals’ method and ComBat, and to 6.5% after SVA. The PC-PR2 approach applied on *M* values estimated values of partial R^2^ for laboratory factors and sample characteristics similar to those of *β* values.

**Fig. 3:**
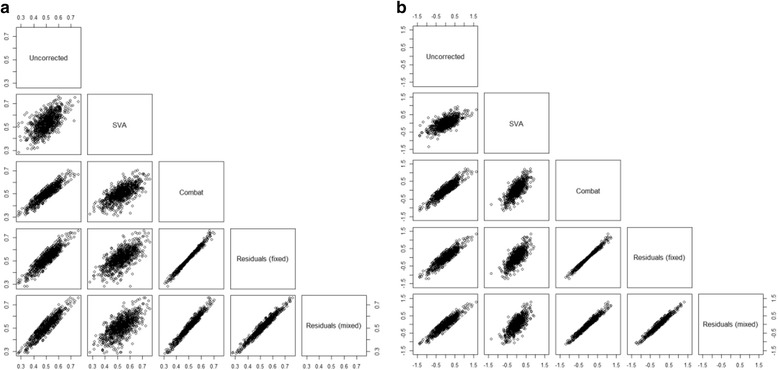
DNA methylation levels of the CpG site cg00000029 before and after normalization step. **a** *β* values. **b** *M* values

Corrected methylation values of the probe cg00000029 were very similar using ComBat or the residuals’ methods for *β* values and *Μ* values (Fig. 3). SVA corrected values were the corrected values most different from the raw values. Using the residuals’ method with fixed or random effects for batch and row sample position gave similar results.

### CpG site-specific models

The frequency *k* of sites associated with smoking status is shown in Table 3, consistently for *β* and *M* values. For *β* values adjusted by batch and row sample position (standard adjustment), smoking status was significantly associated to methylation levels in 444 sites. The number of CpG sites significantly associated with smoking status was equal to 427 for the residuals’ method, 600 for ComBat and 96 for SVA after correction. According to the inflation factors and QQ plots, there was no evidence of inflation for any methods (Additional file 2: Figure S2).

**Table 3:** CpG site-specific regression models before and after normalization step

Values	Methods	Significant sites^b^	CHARGE^c^	Sensitivity	1-Specificity
*β* values	Standard adjustment^a^	444	357 (80%)	1.9×10^−2^	2.2×10^−4^
	Residuals	427	365 (85%)	1.9×10^− 2^	1.5×10^− 4^
	ComBat	600	411 (69%)	2.2×10^− 2^	4.7×10^− 4^
	SVA	96	89 (92%)	0.5×10^−2^	0.2×10^−4^
*M* values	Standard adjustment^a^	322	274 (85%)	1.5×10^−2^	1.2×10^−4^
	Residuals	332	299 (90%)	1.6×10^−2^	0.8×10^−4^
	ComBat	387	335 (87%)	1.8×10^−2^	1.3×10^−4^
	SVA	144	134 (93%)	0.7×10^−2^	0.2×10^−4^

Models are adjusted for chip position, recruitment centre, the five percentages of leukocyte subtypes and age at recruitment, menopausal status and BC status

^a^Also adjusted for batch and sample position

^b^Number of significant sites for smoking status after *p* values FDR correction

^c^Number (and percentage) of significant sites identified by the CHARGE meta-analysis

**Fig. 4:**
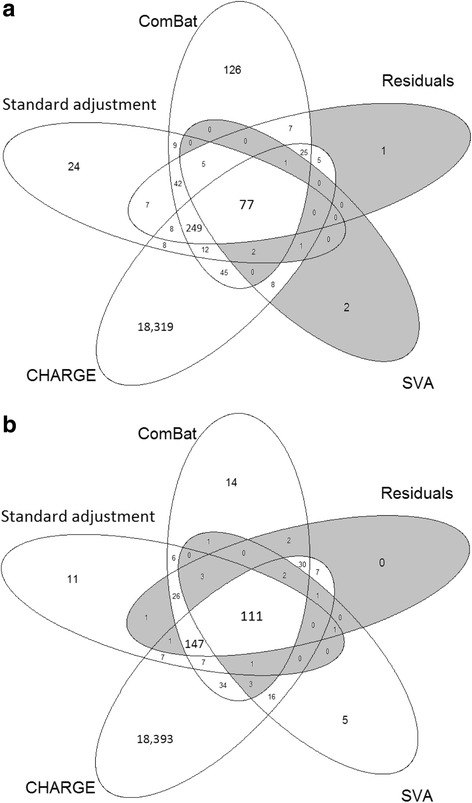
Venn diagram of significantly identified CpG sites for smoking status using each correcting methods and CHARGE. **a** *β* values. **b** *M* values. *p* values were corrected for multiple testing with FDR

These frequencies were compared to the list of 18,760 sites identified in the CHARGE meta-analysis (Joehanes et al. [4]). A total of 77 sites overlapped across the standard adjustment and the three correcting methods in this study and the sites identified in the consortium, as shown in the Venn diagram for *β* values in Fig. 4a. In addition to these sites, the standard adjustment, the residuals’ method and the ComBat method shared a list of 249 significant sites with CHARGE. The ComBat method resulted in the largest frequency of sites overlapping with results in CHARGE (*k* = 411), but also in the largest percentage of sites not observed in CHARGE (31%). In contrast, SVA identified the lowest number of significant sites (*k* = 96) but the vast majority of them (92%) were also identified in CHARGE.

As for *M* values, 322 sites were associated to smoking using the standard adjustment, *k* = 332 after the residuals’ method, *k* = 387 using ComBat, *k* = 144 after SVA correction. A total of 111 sites overlapped all the methods and CHARGE, as shown in Fig. 4b. SVA was the method leading to the lowest number of significant sites, but also to the largest percentage of sites also identified by CHARGE (93%). This percentage ranged between 85 and 90% for all the other methods. According to the inflation factors and QQ plots, there was no evidence of inflation for any methods for *M* values (Additional file 3: Figure S3). SVA showed the least inflation in both *β* values and *M* values.

Sensitivity was similar for the standard adjustment, the residuals’ method and the ComBat method with a value about 0.020 for *β* values and over 0.015 for *M* values (Table 3). SVA sensitivity was four times less for *β* values and twice less for *M* values. SVA was the most specific method with 1-specificity equals to 0.2×10^− 4^ for *β* values and *M* values whereas ComBat was the least specific with 1-specificity equals to 4.7×10^− 4^ and 1.3×10^− 4^ for *β* values and *M* values, respectively.

## Discussion

Batch effects on DNA methylation measurements have already been documented [13]. Various correcting methods have been recently used, including standard adjustment [3], ComBat [6] and SVA [2]. Our findings suggested that batch was not the only source of variation in the DNA methylation data from our EPIC study, as the position of the sample within the chip and, to a lesser extent, chips within batches, also contributed to total variability. Noteworthy, while variation by batch was essentially random, the position of the sample within the chip contributed systematic variation, with methylation levels progressively increasing by row, but not by column. This might be due to the washing step which is done row by row in each chip during the measurement of DNA methylation using HM450K. Eventually, batch and row sample positions explained cumulatively more than 20% of the methylation levels and were the most important sources of variation. Further replications are needed in others dataset from other labs to validate our findings.

PC-PR2 is a powerful method to identify and quantify random and systematic sources of variation in large-scale datasets. Here, the method, initially developed for metabolomics data [17], was successfully applied to epigenetics data, a challenging set characterized by hundreds of thousands of features, and can easily be extendable to other *-omics* data. It is based on the combination of a principal component analysis (PCA) and the concept of partial R^2^ in multivariable linear regression. PC-PR2 quantifies the contribution of variability of continuous and/or categorical covariates to total variability in the outcome data, and in general offers high level of flexibility to capture specific features such as, say, non-linear effects and longitudinal data. A particularly appealing feature is the possibility of performing PCA by decomposing the matrix *XX*^′^ of dimension *n* × *n* rather than *X*^′^*X* of dimension *p* × *p* that would be virtually untreatable in the *-omics* domain. The PC-PR2 can also be extended to the Infinium MethylationEPIC BeadChip (850K), which is the updated version of HM450K.

Identifying unwanted sources of variation in epigenetics data is a crucial step prior to statistical analysis. Each of the three tested methods succeeded to correct DNA methylation levels for the pre-specified sources of variability. Percentages of variability due to batch and row sample position diminished to marginal levels after the use of the three methods. Other unknown or unmeasured experimental conditions are also likely to modify DNA methylation measurements, such as differences in sample handling and preparation and the room temperature during sample processing. Overall, the procedures for sample treatment are way more challenging to control, possibly because detailed information on each sample are not always documented, and it is rather assumed that these are relatively homogeneous across recruitment centres. Statistical adjustment for centre is a standard practice in the analysis of epigenetics data and of any laboratory measurements. In this respect, SVA turned out to provide a correction on top of the pre-specified sources of variability through the estimation of surrogate variables possibly influencing overall variability. It was remarkable that the variability attributed to chip position, whose partial R^2^ values was 6.5% in the raw data, decreased to 0.9% after SVA, even if chip position was not included in the list of covariates of which we want to remove the variability, specified in the SVA model. Indeed, the surrogate variables, computed by a PCA step in the SVA algorithm, capture the variability in the methylation data which is not already explained by the a priori list of covariates (batch and row sample position). A challenge of DNA methylation data is the presence of outliers that can generate spurious associations. Techniques have been introduced to filter out outliers through preliminary quality control checks globally on all CpG sites [32]. This was achieved through the Illumina GenomeStudio quality in the present study [22]. Nevertheless, outlier values passed the GenomeStudio quality control screening and were detected after applying the residuals or SVA methods. On the contrary, ComBat is based on an empirical Bayesian procedure with an additive and a multiplicative component, the latter contributing to shrink all observations, including outliers [14]. This makes ComBat an attractive solution to control outlier values in large-dimension data. Another interesting feature is that ComBat preserved the observed variability of methylation data in the [0, 1] interval for *β* values, unlike the residuals’ and SVA methods, for which the corrected values could fall outside the [0, 1] range.

The performance of the various correction methods was evaluated in this study through the comparison with results of association between smoking and methylation from the CHARGE consortium, one of the largest studies available to date. This could be a debatable choice but allowed a reference group to be established to compute relative sensitivity and specificity of each normalizing method. The low sensitivity across all methods in our analysis might be explained by the lack of power due to the sample size: over 16,000 samples were included in CHARGE against 902 in our study. Some different characteristics of our population and the one of the CHARGE consortium might also explain the difference in terms of significant sites. For example, only women are included in our analysis and half of them developed latter a breast cancer. This makes more difficult the identification of false positives based on the results from the CHARGE consortium. The analysis showed that ComBat had the highest level of relative sensitivity, i.e. relatively less false negative CpG associated to smoking, compared to the residuals and SVA, consistently for *β* or *M* values. On the other hand, SVA came across as the method with, by far, the highest specificity, possibly indicating lesser predisposition to the commit of false positives. As SVA made a much more aggressive correction of systematic variability, the sites identified by SVA are more likely to be universal disruption due to smoking which can explain its higher specificity and its lower sensitivity. In order to avoid over-adjustment using SVA, latent covariates related to subgroups such as the chip position should not be included in the regression model. SVA outperformed both the residuals and, in particular, ComBat, whose lack of specificity turned out to be substantial. In research domains characterized by the danger of populating the scientific literature with false positive findings, like in the *-omics* era, the performance of SVA towards conservative results was deemed to be a valuable feature. Our results would need to be replicated in another dataset.

The *β* values are approximations of the percentage of methylation in a CpG site. Their distribution is often skewed and ranged from 0 to 1. On the other hand, *M* values approximate a normal distribution but are more complex to interpret, as they do not have an obvious biological meaning. It has been recommended to use *M* values for conducting methylation analysis and to use the *β* values when reporting results due to their intuitive biological interpretation [26]. In our study, the PC-PR2 method identified the same sources of variability explaining a similar amount of the total variability using *M* or *β* values. This is likely a consequence on the fact that PC-PR2 is a descriptive method that does not use statistical inference. The association between smoking and DNA methylation was slightly attenuated in terms of number of significant sites using the *M* values, rather than *β* values, for the standard adjustment, residuals’ correction and ComBat correction. Only SVA identified more significant sites with the *Μ* values. *β* values were more sensitive but less specific than *M* values, i.e. more significant sites, including both true and false positive sites.

Approaches for correcting batch effects have been compared using microarray data of gene-expression profiles [33]. In that study, a parametric prior ComBat and a non-parametric ComBat were compared to SVA and to three other methods, including distance-weighted discrimination [34], mean-centering [35] and geometric ratio-based [36] methods. Using two microarray datasets from brain RNA samples and two simulated datasets, ComBat outperformed overall the other methods. In particular, both parametric and non-parametric ComBat algorithms allowed a better control of the variation attributed to batch effect and a better increase of Pearson’s correlation coefficient of the replicates in the microarray data and determined the largest AUC in their assessment of overall performance.

ComBat has also been compared to six other methods to correct for batch effect in microarray data [37], including Deming regression [38], Passing-Bablok regression [39], linear mixed model, a third-grade polynomial regression, the non-linear Qspline method [40] and the ReplicateRUV approach [41]. The first five methods calculate residuals based on different regression models. ReplicateRUV removes unwanted variation based on negative control genes and sample replicates. The combination of quantile normalization and ComBat in large-scale gene expression data in the Gutenberg Health Study removed batch effect and preserved biological variability [37].

In this work, we chose to focus on the residuals, ComBat and SVA approaches, because they are the currently most common methods used to remove unwanted variation in DNA methylation. This work can also be applied to the newer methods which are recently available such as the Bacon approach, a Bayesian method to control bias and inflation in EWAS and TWAS based on estimation of the empirical null distribution [42].

## Conclusions

Our results suggest that in order to reduce the contribution to systematic variation of DNA methylation, it is essential to randomly allocate samples within chips and batches. This is particularly relevant in nested studies for case-control pairs, possibly within the same row position within a chip. We have shown that the PC-PR2 method on DNA methylation levels lent itself as a very useful tool to explore an a priori list of laboratory factors and sample characteristics and to identify the ones possibly determining unwanted variability in large-scale dimension sets such as epigenetics data. This step turned out to be essential to guide the choice of correcting methods, such as the regression-based residuals, ComBat or SVA, and to further appreciate the extent of these corrections. These steps should be part of the pre-processing analysis of any *-omics* data. SVA should specifically be considered when sources of variability are not known. ComBat and the residuals’ method require that potential sources of variability are identified.

## Additional files

Additional file 1: **Figure S1.** Box plots of global methylation (*M* values) according to laboratory factors: batch (a), chip position within batches (b), sample position within chips (c). (PDF 99 kb)

Additional file 2: **Figure S2.** Quantile-quantile (QQ) plots for CpG site-specific analysis with respect to smoking using standard adjustment (a), residuals (b), ComBat (c) and SVA (d) correcting methods for the *β* values. The inflation factor *λ* is defined as the ratio of the median of the observed log_10_ transformed *p* values from the CpG site-specific analysis and the median of the expected log_10_ transformed *p* values. (PDF 110 kb)

Additional file 3: **Figure S3.** Quantile-quantile (QQ) plots for CpG site-specific analysis with respect to smoking using standard adjustment (a), residuals (b), ComBat (c) and SVA (d) correcting methods for the *M* values. The inflation factor *λ* is defined as the ratio of the median of the observed log_10_ transformed *p* values from the CpG site-specific analysis and the median of the expected log_10_ transformed *p* values. (PDF 110 kb)

## Abbreviations

BC: Breast cancer
EPIC: European Prospective Investigation into Cancer and nutrition
FDR: False discovery rate
HM450K: Illumina Infinium HumanMethylation450K
PC-PR2: Principal component partial R-square
SVA: Surrogate variables analysis
