# Supplementary figures and images for: Identifying and correcting epigenetics measurements for systematic sources of variation
Source: Clin Epigenetics. 2018 Mar 21;10:38. doi: 10.1186/s13148-018-0471-6 (PMC5863487; doi:10.1186/s13148-018-0471-6)

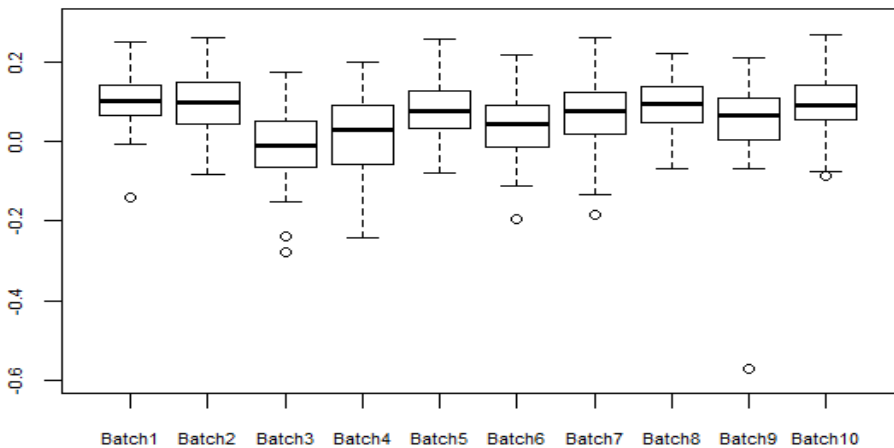

(a)

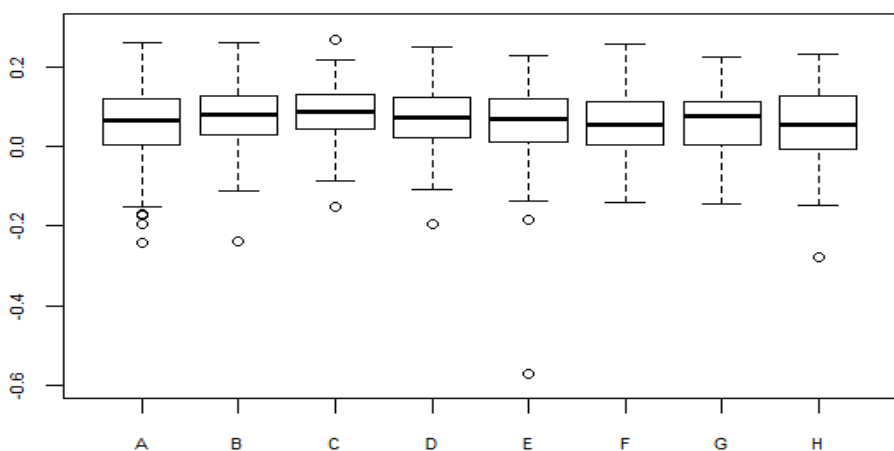

(b)

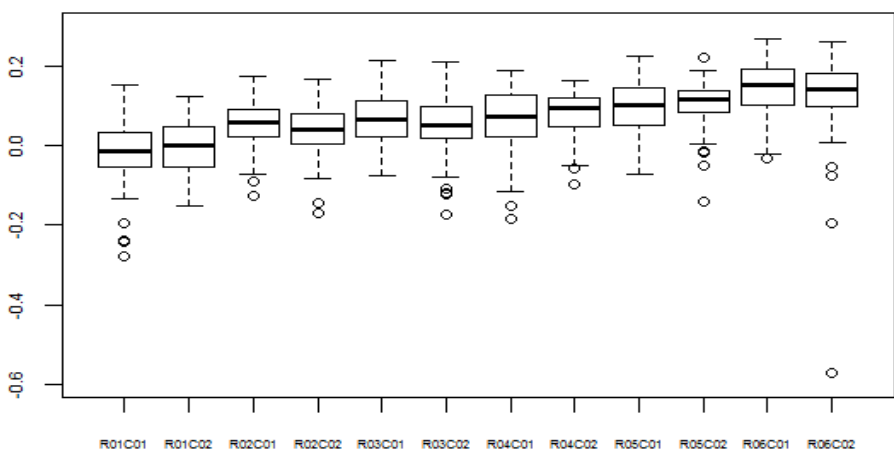

(c)

Supplement: Supplementary file 1 — Figure S1. Box plots of global methylation (M values) according to laboratory factors: batch (a), chip position within batches (b), sample position within chips (c). (PDF 99 kb) [file 13148_2018_471_MOESM1_ESM.pdf]

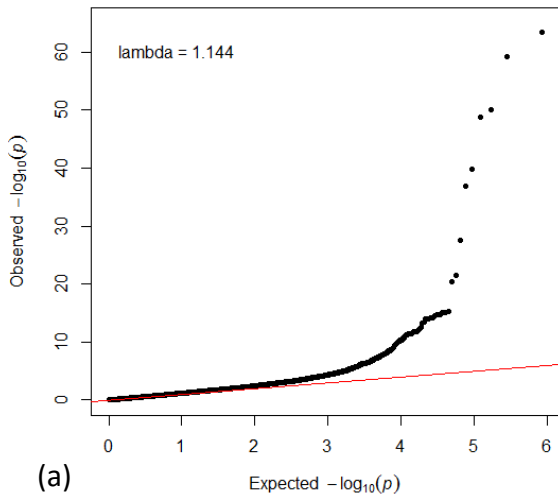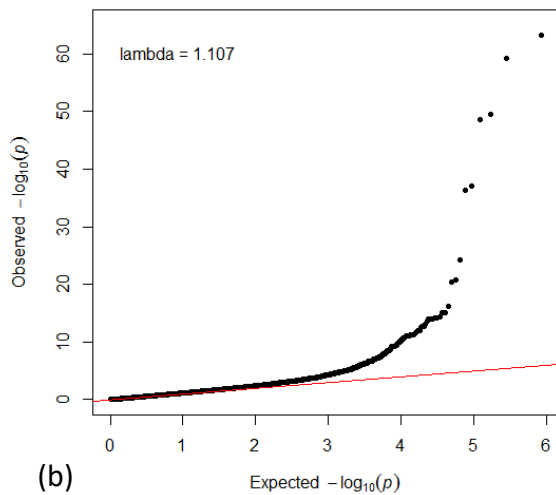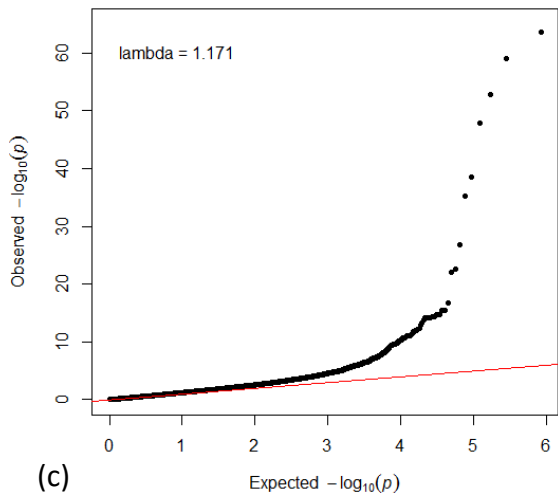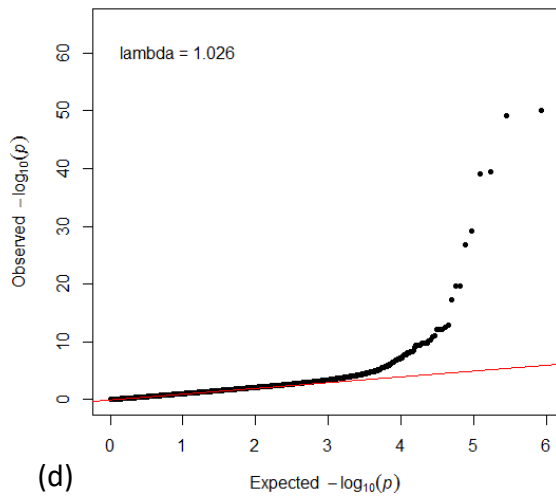

Supplement: Supplementary file 2 — Figure S2. Quantile-quantile (QQ) plots for CpG site-specific analysis with respect to smoking using standard adjustment (a), residuals (b), ComBat (c) and SVA (d) correcting methods for the β values. The inflation factor λ is defined as the ratio of the median of the observed log10 transformed p values from the CpG site-specific analysis and the median of the expected log10 transformed p values. (PDF 110 kb) [file 13148_2018_471_MOESM2_ESM.pdf]

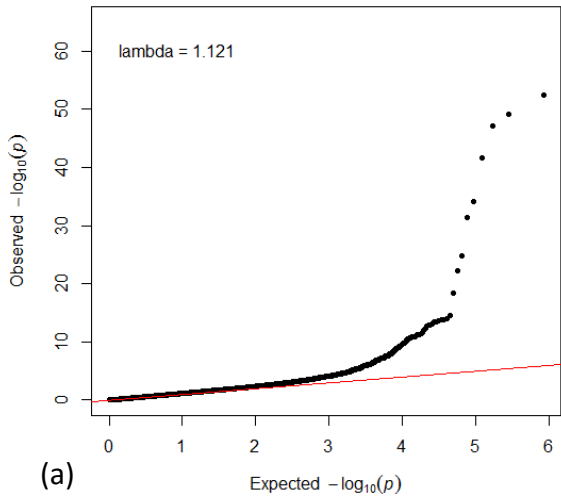

(a)

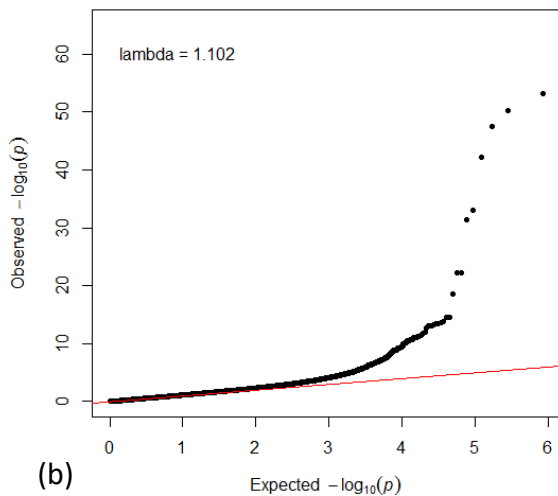

(b)

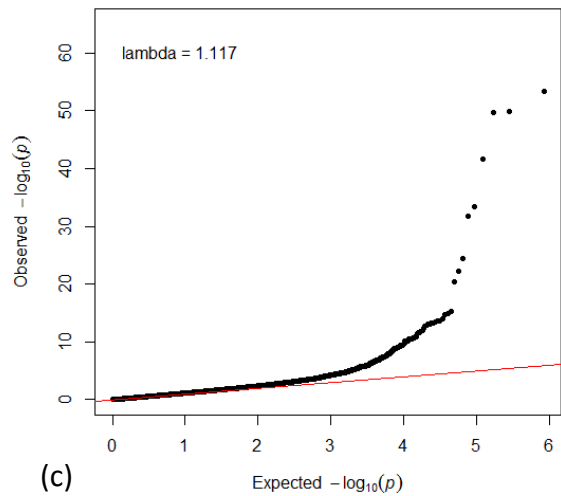

(c)

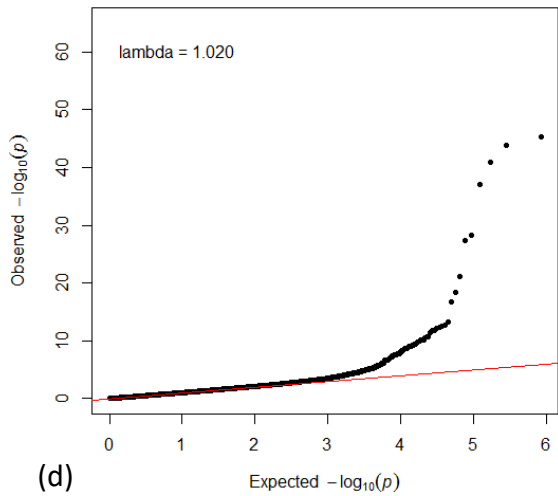

(d)

Supplement: Supplementary file 3 — Figure S3. Quantile-quantile (QQ) plots for CpG site-specific analysis with respect to smoking using standard adjustment (a), residuals (b), ComBat (c) and SVA (d) correcting methods for the M values. The inflation factor λ is defined as the ratio of the median of the observed log10 transformed p values from the CpG site-specific analysis and the median of the expected log10 transformed p values. (PDF 110 kb) [file 13148_2018_471_MOESM3_ESM.pdf]
